# A planetary health perspective on the climate crisis

**DOI:** 10.25646/10389

**Published:** 2022-08-31

**Authors:** Sabine Gabrysch

**Affiliations:** Potsdam Institute for Climate Impact Research, Research Department Climate Resilience Charité – Universitätsmedizin Berlin, Institute of Public Health

Planetary Health is about the health of humans and other living beings, of populations and ecosystems, and of the whole living planet Earth, appreciating the deep connectedness of all life.

A planetary doctor examining the Earth would find three core symptoms: 1.) the climate heating up, 2.) pollution of air, water and land and 3.) a massive loss of biodiversity. The causative agents are humans who have multiplied their population size and resource use over a short time.

Human living conditions have improved substantially in many parts of the world over the last decades, and so has human health. However, the poorest have been left behind and modern lifestyles have led to new health problems: Too much and unhealthy food, too little exercise and air pollution are causing a sharp rise in obesity, diabetes and cardiovascular disease worldwide. In addition, there are new health risks due to human interventions in nature [1]: Climate change is intensifying weather extremes with deadly heat waves and floods. Droughts cause harvest failures and famines. The destruction of natural habitats makes pandemics more likely.

The diagnosis is serious: This is a planetary emergency. We are transgressing several planetary boundaries and getting close to dangerous tipping points in the Earth system. Humanity is destroying living conditions on the planet, risking its habitability and the survival of our civilisation. It is also a justice issue: The rich are causing the problem and the poor and future generations bear the brunt.

In an emergency one has to act fast and courageously. How can we achieve a radical reshaping of our whole way of living, our way of doing business, a Great Transformation of society at record pace? An unprecedented challenge.

It will require major shifts in our worldview, our relationship to nature, our values [2]. We need to realise that we humans are one component of a highly complex web of life, one part of our living planet, and that we totally depend on it. Our health, our society, our economy all depend on functioning ecosystems, on a stable climate. We need to regain a wisdom many indigenous peoples have preserved over generations.

The good news is that tackling this challenge could be a huge global health opportunity. Plenty of solutions are synergistic: good for the planet and good for our health. A win-win situation! For example, to stop burning fossil fuels can avoid millions of deaths from air pollution. Making cities easy to walk and bike helps people exercise more. And eating less meat and more vegetables would be a huge win for climate, biodiversity, animal and human health.

The fundamental societal change that we need will not happen by itself or from above. It requires many active people pushing for it. Jointly, we can change the structures that are hurting people and nature. We can create a positive vision for a better, healthier, sustainable world which already exists in niches.

As health professionals, we have a particular responsibility to protect health, and also a high credibility. We can play an important role in the public debate on the planetary crisis [3], by warning of the risks, but also pointing to the long-term and short-term health gains.
